# Seroprevalence of Chikungunya Virus after Its Emergence in Brazil 

**DOI:** 10.3201/eid2409.180724

**Published:** 2018-09

**Authors:** Patrick Gérardin, André Ricardo Ribas Freitas, Daouda Sissoko

**Affiliations:** Centre Hospitalier Universitaire Réunion, Saint Pierre, France (P. Gérardin);; Unité Mixte 134 Processus Infectieux en Milieu Insulare Tropical, Sainte Clotilde, France (P. Gérardin);; San Leopoldo Mandic School of Medicine, Campinas, Brazil (A.R.R. Freitas);; Biomerieux Africa Cluster, Abidjan, Côte d’Ivoire (D. Sissoko)

**Keywords:** alphavirus, asymptomatic infection, Brazil, chikungunya virus, chronic arthralgia, cross-sectional study, geographic information systems, parasites, prevalence, population-based study, seroprevalence, serosurvey, vector-borne infections, viruses

**To the Editor:** In their well-designed and timely serosurvey, Dias et al. provide evidence of high prevalence of East/Central/South African (ECSA) genotype chikungunya virus (CHIKV) (seropositivity rate 51.0%) in population subsets of 2 urban communities in Bahia state, Brazil (*1*). The authors found a high proportion of asymptomatic CHIKV patients (63.2%; 268/424). In addition, the prevalence of chronic arthralgia among infected persons (26.4%; 112/424) was lower or within the expected range of previously reemerging clades of CHIKV, namely ECSA diverged (Indian Ocean lineage) or Asian lineage, respectively. However, a high proportion of the symptomatic participants in Dias et al. reported chronic symptoms lasting >3 months (71.8%; 112/156). We comment on these findings. 

First, Dias et al. report that the selected locations were at the epicenter of the transmission area and, therefore, the data cannot be extrapolated to other cities. To better understand the dynamics of the disease, it would be useful to select locations more representative of other infected areas. 

Second, the modest participation at the study locations (66.5%; 831/1250) could be related to using the more painful venipuncture method instead of a fingerstick to draw blood. In comparison, the participation rate was ≈80% in a Réunion Island serosurvey for CHIKV for which the fingerstick method was used (*2*). The participation level suggests the possibility of self-selection bias toward infected patients, who might be more interested in knowing their serologic status. Self-selection bias might explain the high proportion of symptomatic patients who self-reported having a chronic form of chikungunya. 

Last, because of their unreliable discriminatory performance, using fever and arthralgia to identify symptomatic patients might have increased the proportion of patients misclassified as asymptomatic (i.e., patients with symptoms other than fever and arthralgia being falsely classified as negative) (*3*). These limitations being specified, the prevalence of chronic arthralgia among symptomatic patients in Dias et al. falls within the expected range of the Asian lineage of CHIKV, the other clade circulating in the Americas (*4*,*5*), which confers external validity to the study.
